# Sensitivity enhanced tunable plasmonic biosensor using two-dimensional twisted bilayer graphene superlattice

**DOI:** 10.1515/nanoph-2022-0798

**Published:** 2023-03-07

**Authors:** Fusheng Du, Kai Zheng, Shuwen Zeng, Yufeng Yuan

**Affiliations:** School of Electronic Engineering and Intelligentization, Dongguan University of Technology, Dongguan, 523808, China; School of Civil Aviation, Northwestern Polytechnical University, Xi’an, Shanxi, 710072, China; Light, Nanomaterials & Nanotechnologies (L2n), CNRS-ERL 7004, Université de Technologie de Troyes, Troyes, 10000, France

**Keywords:** GH shift, human hemoglobin, SARS-CoV-2, sensitivity enhancement, tunable plasmonic biosensor, twisted bilayer graphene superlattice

## Abstract

This study theoretically demonstrated an insight for designing a novel tunable plasmonic biosensor, which was created by simply stacking a twisted bilayer graphene (TBG) superlattice onto a plasmonic gold thin film. To achieve ultrasensitive biosensing, the plasmonic biosensor was modulated by Goos–Hänchen (GH) shift. Interestingly, our proposed biosensor exhibited tunable biosensing ability, largely depending on the twisted angle. When the relative twisted angle was optimized to be 55.3°, such a configuration: 44 nm Au film/1-TBG superlattice could produce an ultralow reflectivity of 2.2038 × 10^−9^ and ultra-large GH shift of 4.4785 × 10^4^ µm. For a small refractive index (RI) increment of 0.0012 RIU (refractive index unit) in sensing interface, the optimal configuration could offer an ultra-high GH shift detection sensitivity of 3.9570 × 10^7^ µm/RIU. More importantly, the optimal plasmonic configuration demonstrated a theoretical possibility of quantitatively monitoring severe acute respiratory syndrome coronavirus (SARS-CoV-2) and human hemoglobin. Considering an extremely small RI change as little as 3 × 10^−7^ RIU, a good linear response between detection concentration of SARS-CoV-2 and changes in differential GH shift was studied. For SARS-CoV-2, a linear detection interval was obtained from 0 to 2 nM. For human hemoglobin, a linear detection range was achieved from 0 to 0.002 g/L. Our work will be important to develop novel TBG-enhanced biosensors for quantitatively detecting microorganisms and biomolecules in biomedical application.

## 1 Introduction

Two-dimensional (2D) twisted bilayer nanostructures are a class of nanoscale configurations, whose electronic, photonic, and plasmonic properties highly depend on a twisted angle between two superimposed layers of van der Waals (vdWs) materials. It is worth noting that, those stacked configurations in nature are usually artificial, and they can be fabricated by chemical vapor deposition approach [1]. To date, typical 2D bilayer configurations such as TBG [2–4], twisted bilayer transition metal dichalcogenides (TMDs) [5, 6], twisted bilayer black phosphorus (BP) [7, 8], and twisted bilayer hexagonal boron nitride (h-BN) [9], have been proposed by theoretical prediction and experimental fabrication. Compared with monolayer 2D material, bilayer 2D stacking configurations exhibited novel electronic, photonic, and plasmonic features. The reason is that, it can form a moiré superlattice when two layers are stacked each other with a small rotated angle (*α*) [2, 10].

As the firstly proposed 2D twisted bilayer configuration [2], TBG superlattice has received the most attention because of its exotic features and exploration of promising applications. Considering a bilayer graphene system, there are three stacking forms including AA-, AB-, and twisted mismatching-types [11, 12]. For AA model, the carbon atoms in both upper and lower graphene layers are parallelly stacked together, and there is no misalignment in lateral coordinates of carbon atoms. For AB stacking configuration, one graphene layer is parallelly shifted by a bond length, and there are only half of carbon atoms in two graphene layers contacting each other. Compared to parallel misalignment in lateral coordinates, twisting exhibits significant advantages, providing a new freedom degree to understand interlayer coupling in a bilayer graphene system. Using twisting, it is possible to change the electronic properties of a bilayer graphene structure without destroying its material composition. Through changing a relative in-plane twisted angle, a distinguished TBG superlattice can be yielded. The electronic, photonic and plasmonic properties in a TBG system are significantly different from other two stacking configurations (AA and AB). Moreover, they are tunable by varying the twisted angle (*α*). It means that, they can work as a function of twisted angle (*α*). Therefore, these unique features make TBG superlattice extremely interesting and motivated many scientists to study TBG.

In light of significant changes in electronic properties of TBG superlattice generated by a small physical tweak, a new research field named “twistronics” to study how the tunable twisted angle can change the electronic properties of stacked 2D material layers has been established [13, 14]. Herein, a host of interesting electronic phenomena have been reported using TBG configuration, such as correlated insulators [4, 15], strong electronic correlations [16, 17], unconventional superconductivity [18, 19], ferromagnetism [20, 21], Josephson effect [22], and nanolaser with a high figure of merit [23]. Meanwhile, the special tunable twisted angle also changes the optical and plasmonic properties of TBG system, showing great potential in designing novel twisttronic devices. For example, Xin et al. [24] studied the photovoltage enhancement response in a TBG configuration using surface plasmon resonance (SPR) technology. It showed that, the optical absorption in the TBG system highly depended on the twisted angle. With help of strong photon absorption in TBG system, the SPR effect can significantly enhance the photon response. This study provided a great possibility to incorporate surface plasmons polaritons (SPPs) with 2D TBG nanostructure for designing new plasmonic sensor.

Compared to plasmonic metallic thin film such as gold and silver, graphene plasmons have showed ultra-high electromagnetic field confinement effects, making it extremely sensitive to the variation of local dielectric environments [25]. Similar with monolayer layer graphene, the collective behavior of SPPs wave oscillating in TBG is still from charge excitation mechanism. To date, there are four plasmon models in TBG system, such as transverse plasmons, inter-band plasmon, damped plasmons, and undamped plasmons [26]. It has been reported that, when the twisted angle is relatively small, the wavelength of SPPs in TBG is identical to single layer graphene. However, as the twisted angle become larger, the wavelength of SPPs in TBG system increases significantly [27]. Different twisted angles can generate different electronic states employed for interlayer electron coupling, resulting in plenty of SPR excitation conditions. These above observations showed that, the twisted angle in TBG system can highly affect the excitation of SPPs. Therefore, TBG-based configuration has great potential in creating novel plasmonic devices. The refractive indices of TBG system under different twisted angles are the most important indicators employed for sensitivity development and optimization of plasmonic devices. Recently, Kumar et al. [11] theoretically calculated the refractive indices of TBG configuration under various twisted angles. Such important parameters are extremely useful for designing TBG-based plasmonic biosensor. It is well-known that, graphene-based configurations have been employed in performing ultrasensitive biosensing [28]. Typically, the photon absorption in a TBG superlattice can be significantly manipulated via tuning a planar twisted angle. Herein, twisting can provide a new insight into exploring tunable plasmonic biosensors. However, there is a few reports on creating novel SPR biosensor using a TBG superlattice.

Motivated by above stated observations, a novel SPR biosensor was theoretically proposed using a TBG superlattice in our study. It is worth noting that, compared with other reported graphene-based metasurfaces [29–31], our proposed plasmonic configuration has the advantages of simple fabrication and low lost. It was a simple vdWs stacking heterostructure consisting of a plasmonic Au film, and a TBG superlattice. Under the excitation of p-polarized light at 632.8 nm, the plasmonic biosensor was created by calculating the lateral position shift named GH shift, exhibiting ultrasensitive detection performance. More interestingly, our proposed biosensor showed a tunable biosensing performance via changing the twisted angle (*α*). When the twisted angle was fixed at 55.3°, an ultra-low reflectivity of 2.2038 × 10^−9^ was yielded by the best configuration: 44 nm Au film/1-TBG superlattice. It indicated that, almost 100% photon energy was employed to support SPR enhancement. Through deriving the phase of reflection photon, the largest GH shift was determined to be 4.4785 × 10^4^ µm. When the optimal configuration was employed to measure a fixed RI change in 0.0012 RIU, it provided an extremely high GH shift sensitivity of 3.9570 × 10^7^ µm/RIU. Furthermore, the optimal biosensor configuration was employed to theoretically monitoring the detection of targeted bioanalytes including SARS-CoV-2 and human hemoglobin for a slight RI variation as little as 3 × 10^−7^ RIU. A good linear response between detection concentration of targeted bioanalytes and changes in differential GH shift were obtained. It is important that, Au film/TBG superlattice heterostructure has great potential in quantitatively monitoring biological samples in practical applications.

## 2 Methodology

To date, there are several major approaches employed for exciting SPPs, such as prism-, waveguide-, and grating-coupling. Compared with other two coupling approaches, prism-coupling model is the most conventional approach via integrating a prism coupler with a plasmonic metal film. Thus, it can easily control the parameter and variable, because of its alignment simplicity. The sensing principle is that, using p-polarized light to excite and conduct the SPPs wave in thin metal film via a high RI prism. Moreover, the RI of prism should be larger than the RI of sensing buffer. Under wave vector matching conditions, the attenuated total reflectance (ATR) occurs. Then, it will generate a SPR curve through measuring the reflection spectra.

**Figure 1:**
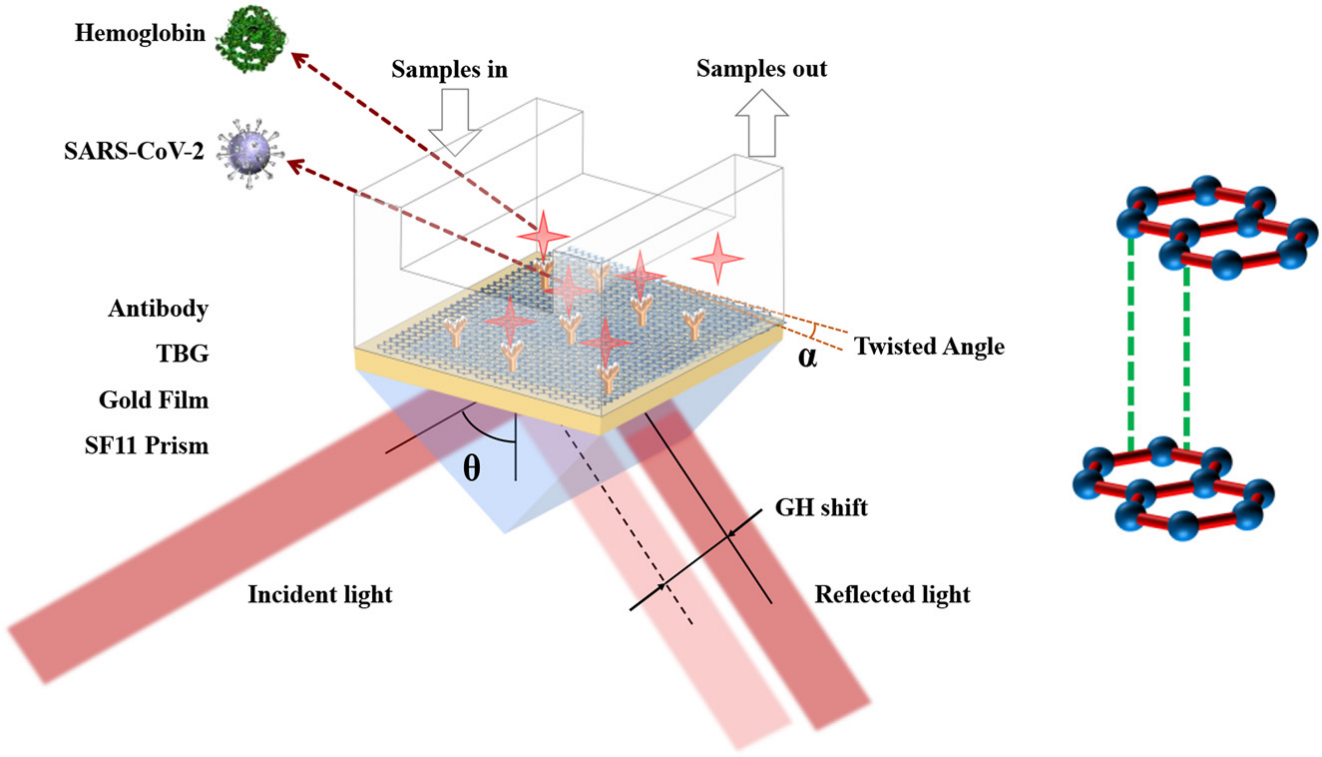
Schematic diagram of TBG-enhanced SPR biosensor.

The sensitivity enhanced plasmonic biosensor was composed of a SF11 prism, a gold thin film, and a TBG superlattice with a tunable twisted angle (*α*), as depicted in Figure 1. In our study, the Kretschmann prism configuration was adopted to excite the SPPs wave in gold film-TBG heterostructure. The incident light was a 632.8 nm p-polarized light, and the reflected light experienced a sharp phase jump under ATR condition. Owing to significant displacement behavior between incident light and reflected light, GH shift was employed to evaluate SPR enhancement effects. Although silver exhibits sharper SPR reflection band, gold shows better sensitivity and bandwidth. In addition, gold exhibits higher surface stability than silver. In brief, the plasmonic gold film was efficiently attached to the upper surface of SF11 prism through refractive index matching liquid. In plasmonic optics, plasmonic gold film usually has large ohmic energy loss, and the transmission distance of SPPs wave is very limited. It is beneficial to introduce a low-loss plasmonic material to improve transmission distance in gold film. The high carrier mobility in plasmonic material is important to guarantee low energy dissipation in plasmonic sensing device [32]. It is well-known that, graphene is the most extensively studied star in 2D family. Compared to other members such as TMDs, BP, and h-BN, suspending monolayer graphene has an ultra-high carrier mobility of 2 × 10^5^ cm^2^ V^−1^ s^−1^ [33]. When monolayer graphene is deposited on plasmonic gold thin film, strong coupling can be generated at the gold film-graphene interface, because of strong charge transfer between graphene and gold film, further making plasmonic gold-graphene heterostructure with the ability of ultrasensitive biosensing. Eventually, a highly enhanced electric field enhancement will be produced at the gold film-graphene sensing interface. Compared to monolayer graphene system, TBG superlattice can provide a new degree of freedom to study the plasmonic enhancement via twisting. Herein, a TBG system with a planar twisted angle (*α*) was introduced and superimposed onto Au thin film. In our proposed biosensor configuration, the stacking model of bilayer graphene was AB type with a thickness of 0.68 nm, as depicted in Figure 1. In the upmost layer, it was a sealed container filling with running buffer. The mixture solutions containing 10 mM HEPES and 120 mM NaCl solutions were employed to disperse SARS-CoV-2 sample. When the SARS-CoV-2 in buffer fluid was captured by special antibody sites on TBG sensing interface, the RI variation in sensing interface would be produced. Generally, the parameter of reflected light such as intensity, and phase could be measured as an optical response based on a tiny RI change in sensing interface. In this study, GH shift was extracted to assess the ultrasensitive biosensing ability of Au film-TBG heterostructure.

In plasmonic optics, the optical properties of plasmonic materials are usually evaluated by both the permittivity and permeability. Moreover, the relative permeability of conventional plasmonic materials is not considered in visible and near-infrared regions. The reason is that, the relative permeability is usually regarded to be unity and the magnetic susceptibility is negligible. Then, it will simplify the description of optical RI. Using Fresnel equations, both refraction and reflection features of plasmonic materials can be characterized. The complex permittivity ( $\bar{\varepsilon }$ ) of plasmonic material can be described by $\bar{\varepsilon }=\varepsilon_{1}+\varepsilon_{2}i$ , where the real part *ɛ*_1_ stands for energy absorption in plasmonic material, and the imaginary part *ɛ*_2_ relates to the energy dissipation in plasmonic material. However, the complex RI ( $\bar{n}$ ) of plasmonic material can be defined by $\bar{n}=\sqrt{\bar{\varepsilon }}=\varepsilon +\eta i$ , where *ɛ* and *η* stand for the real part and imaginary part of optical RI, respectively. The imaginary part *η* implies the energy absorption in plasmonic material.

The permittivity of each stacking plasmonic materials largely affected the energy absorption in our proposed configuration. Any variation in permittivity will change its biosensing performance. Then, the optical RI of each stacking layer was determined. Under the illumination of p-polarized incident light at 632.8 nm, the RI of SF11 prism was 1.7786, and the RI of plasmonic gold film 0.1838 + 3.4313i [34]. In addition, the RI of monolayer graphene and AB type-bilayer graphene without twisting was 3.000 + 1.1487i [35], and 2.7411 + 1.4016i [11], respectively. The detailed refractive indices of TBG system with various twisted angles (*α*) were listed in Table 1 [11]. It can be found that, when the incident light is 632.8 nm, the real part of permittivity from plasmonic gold film is negative, resulting in an imaginary part of optical RI. Moreover, both monolayer graphene and TBG superlattice show similar properties with plasmonic gold film. However, TBG superlattice exhibits relatively less energy dissipation and smaller propagation loss.

**Table 1:** optical RI of TBG system with various twisted angles (*α*) under the excitation of 632.8 nm.

Twisted angle	RI	Twisted angle	RI	Twisted angle	RI
0.33°	1.7 + 0.75i	9.32°	2.4 + 0.96i	47.78°	1.15 + 0.5443i
0.48°	1.7 + 0.964i	9.81°	1.7 + 0.41i	55.3°	1.5 + 0.968i
0.76°	0.97 + 0.964i	10°	1.7 + 0.22i	61.54°	3.06 + 1.215i
2.1°	0.97 + 1.1875i	13.31°	1.5 + 0.3355i	73.19°	1.9 + 1.2975i
2.46°	0.97 + 0.75i	21.53°	1.9 + 0.82i	75.29°	1.9 + 1.4684i
2.63°	0.97 + 0.4107i	25.01°	1.9 + 0.968i	76.87°	1.5 + 1.4684i
2.81°	0.97 + 0.2232i	25.96°	1.9 + 1.215i	77.82°	1.5 + 1.6266i
4.08°	0.97 + 0.96i	28.17°	2.57 + 1.215i	81.61°	1.15 + 1.6266i
4.7°	1.7 + 1.19i	31.24°	2.57 + 1.2975i	83.03°	1.15 + 1.4684i
5.26°	2.4 + 1.19i	41.77°	2.57 + 0.968i	84.46°	1.15 + 1.2975i
6.3°	0.97 + 1.19i	45.72°	1.5 + 0.82i	85.88°	1.15 + 1.215i
8.18°	1.7 + 1.38i	46.04°	1.5 + 0.5443i	87.3°	1.15 + 0.968i

It is worth noting that, the thickness of 1 TBG superlattice was 0.68 nm. The RI of buffer mixture was calculated by the following Equation (1):

(1) $$\begin{array}{c} y=0.00004x+1.3341 \end{array}$$

where *y* and *x* are the RI of buffer mixture and concentrations of HEPES solution (mM), respectively. In our study, the concentration of HEPES buffer was assumed to be 10 mM. Based on Equation (1), the RI of running buffer (*n*_*C*_) was determined to be 1.3345 [36]. There was a RI change (Δ*n*) in sensing interface because of special adsorption interactions between SARS-CoV-2 and antibody sites on TBG surface. Moreover, the RI change was proposed to be related with the concentration of SARS-CoV-2, as described by Equation (2) [37]:

(2) $$\begin{array}{c} \Delta n=\left(dn/dc\right)c_{A} \end{array}$$

where *c*_*A*_ is the concentration of SARS-CoV-2. In addition, the coefficient d*n*/d*c* stands for the increment of RI. To study protein–protein interaction, the value of d*n*/d*c* is a constant equal to be 0.186 mL g^−1^ [37].

To study the feasibility of monitoring human hemoglobin, another two buffers including phosphate buffered saline (PBS, 10 mM) and Trizma solutions were employed. In general, the RI of 10 mM PBS solution was determined to be 1.3345 [38], and the RI of 100 mM Trizma solution was determined to be 1.3375 [39]. Using Equation (2), the RI variation in sensing interface caused by adsorption interaction of human hemoglobin can be described. For hemoglobin, the value of coefficient d*n*/d*c* was 0.1423 mL g^−1^ [38].

Four SPR signal-modulation methods including reflectivity, SPR angle (incident angle), differential phase, and GH shift, was further studied via transfer matrix method incorporating with Fresnel theory. Our proposed configuration was a 4-layer model, and each layer was stacked in parallel. In addition, the property of each layer material was homogeneous, nonmagnetic, and isotropic. To guarantee enough adsorption sites, the number of adsorption sites on TBG surface was larger than the number of targeted analytes.

It is well-known that, transfer matrix method is a useful approach to deal with propagation of SPPs waves through N-layer mediums. Based on Maxwell’s theory, when electromagnetic wave propagates across two different mediums, simple continuity conditions need to be satisfied. In a N-layer configuration, if the incident electromagnetic wave is already known at the lower interface of a given layer, the obtained electromagnetic wave at the upper interface of the given layer can be determined by a simple matrix transfer. Then, the stacking N-layers can be described as a system matrix. Finally, the system matrix will be converted into output of reflection coefficients.

For example, we introduce a transfer *M* standing for propagation of p-polarized light through N-1 layers, which can be determined by the following equation:

(3) $$\begin{array}{c} M=\overset{N-1}{\underset{k=2}{\prod }}M_{k}=\left[\begin{array}{cc} M_{11} & M_{12}\\ M_{21} & M_{22} \end{array}\right] \end{array}$$

where *M*_*k*_ can be represented as:

(4) $$\begin{array}{c} M_{k}=\left[\begin{array}{cc} \cos\beta_{k} & \left(-isin\beta_{k}\right)/q_{k}\\ -iq_{k}\sin\beta_{k} & \cos\beta_{k} \end{array}\right] \end{array}$$

Moreover, the two parameters *β*_*k*_ and *q*_*k*_ can be calculated by the Equations (5) and (6):

(5) $$\begin{array}{c} \beta_{k}=\frac{2\pi d_{k}}{\lambda }{\left(\varepsilon_{k}-n_{1}^{2}\sin^{2}\theta_{1}\right)}^{\frac{1}{2}} \end{array}$$

(6) $$\begin{array}{c} q_{k}=\frac{{\left(\varepsilon_{k}-n_{1}^{2}\sin^{2}\theta_{1}\right)}^{\frac{1}{2}}}{\varepsilon_{k}} \end{array}$$

where *k* represents the *kth* superimposing layer. In Equations (5) and (6), *ɛ*_*k*_ is the dielectric constant of *kth* superimposing layer, and *d*_*k*_ stands for the thickness of the *kth* layer. Finally, *θ*_1_ is the incident angle responding to the first layer, and *n*_1_is the RI of SF11 prism through which p-polarized light propagates.

To study the propagation of s-polarized light in various mediums, the above-stated relationships including Equations (3)–(6) are also applicable. However, the parameter *q*_*k*_ should be calculated by Equation (7):

(7) $$\begin{array}{c} q_{k}={\left(\varepsilon_{k}-n_{1}^{2}\sin^{2}\theta_{1}\right)}^{\frac{1}{2}} \end{array}$$

With help of TMM, the total reflectivity (*R*_*p*_) in 4-layer configuration can be determined by Equation (8):

(8) $$\begin{array}{c} R_{p}=r_{p}^{2}={\left|\frac{\left(M_{11}+M_{12}q_{N}\right)q_{1}-\left(M_{21}+M_{22}q_{N}\right)}{\left(M_{11}+M_{12}q_{N}\right)q_{1}+\left(M_{21}+M_{22}q_{N}\right)}\right|}^{2} \end{array}$$

Then, the phase *φ*_*p*_ of p-polarized light can be derived from Equation (9):

(9) $$\begin{array}{c} \varphi_{p}=\arg\left(r_{p}\right) \end{array}$$

To study phase modulation, the differential phase Δ*φ*_*d*_ can be calculated by Equation (10):

(10) $$\begin{array}{c} \Delta \varphi_{d}=\left|\varphi_{p}-\varphi_{s}\right| \end{array}$$

where *φ*_*s*_ is the calculated s-polarized light phase.

In this work, GH shift was selected to assess the biosensing performance of Au film-TBG superlattice configuration. The GH effect is a classical optical coherence phenomenon, which describes polarized light experiences a small lateral displacement behavior in an optical ATR configuration. This GH effect usually occurs at the interface of two different dielectric media, because practical reflection light are finite sized beams rather than ideal monochromatic electromagnetic plane waves. Thus, an optical interference pattern can be generated by two reflected beams. As depicted in Figure 1, the shift between incident light and reflected light is called GH shift, which is perpendicular to the propagation direction of reflected light. Based on stationary phase explanation [40], the theoretical GH shift (GHS) is derived from Equation (11):

(11) $$\begin{array}{c} GHS=-\frac{\lambda }{2\pi }\frac{d\varphi }{d\theta } \end{array}$$

Equation (11) showed that, GH shift is the first-order derivative of phase shift from reflection light. Therefore, GH shift should have sharper singularity than phase shift. It is worth noting that, the GH shift induced by low RI of dielectric medium was extremely small [41, 42]. However, it can be efficiently amplified via SPR effect [34, 43].

Similar with differential phase in Equation (10), the differential GH shift (Δ*GHS*_*d*_) can be obtained by Equation (12):

(12) $$\begin{array}{c} \Delta GHS_{d}=\left|GHS_{p}-GHS_{s}\right| \end{array}$$

According to Equation (12), the differential GH shift (Δ*GHS*_*d*_) between p-polarized and s-polarized light can be considered as an important optical parameter to monitor the concentration of targeted bioanalytes. To measure the differential GH shift, it is necessary to build a setup. Considering He–Ne laser with an output wavelength at 632.8 nm, one He–Ne light beam is separated into two polarized beams (p-polarized light and s-polarized light) by a polarized beam splitter. A SF11 prism is integrated with sensing substrate (Au film-TBG superlattice) with help of index-matching fluid. In a fixed time, an optics chopper is employed to guarantee that only one polarized light beam can be incident onto sensing interface Au film-TBG superlattice. Once the optimal incident angle is fixed, the largest GH shift can be achieved. For various detection concentrations of targeted bioanalytes, the displacement positions between p-polarized light and s-polarized light can be detected using a lateral position detector. Finally, these lateral position signals are extracted using self-compiled Matlab program.

For a small RI variation (Δ*n*_bio_) due to the adsorption interaction between targeted bioanalyte and TBG surface, both phase sensitivity (*S*_*φ*_) and GH shift sensitivity (*S*_*GHS*_) can be calculated by Equations (13) and (14) [44],

(13) $$\begin{array}{c} S_{\varphi }=\frac{\Delta \varphi_{d}}{\Delta n_{\text{bio}}} \end{array}$$

(14) $$\begin{array}{c} S_{\mathrm{GHS}}=\frac{\Delta GHS_{d}}{\Delta n_{\text{bio}}} \end{array}$$

where the phase sensitivity (*S*_*φ*_) with a unit of degree/RIU is defined as the ratio of change in differential phase (Δ*φ*_*d*_) to the small RI variation (Δ*n*_bio_). Similarly, the GH shift sensitivity (*S*_*GHS*_) with a unit of µm/RIU is defined as the ratio of change in differential GH shift (Δ*GHS*_*d*_) to the small RI variation (Δ*n*_bio_).

## 3 Results and discussion

**Figure 2:**
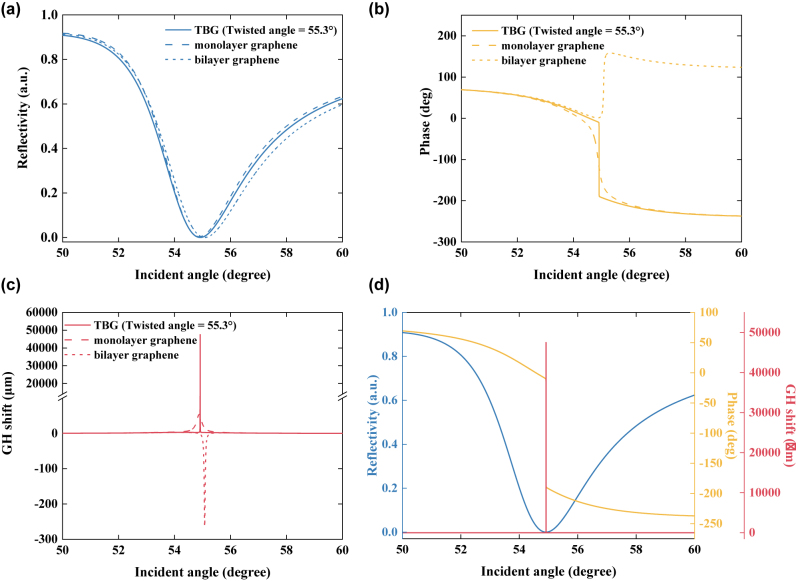
Minimum reflectivity (a), phase (b) and GH shift (c) based on plasmonic Au film-monolayer graphene (dash line), Au film-bilayer graphene without any twisting (short dash line), and Au film-1-TBG superlattice with a twisted angle of 55.3° (solid line). (d) Comparison of minimum reflectivity (blue), phase (orange) and GH shift (red) extracted from Au film-1-TBG with a twisted angle of 55.3°. Noting that, the thickness of Au film is 44 nm, and the excitation wavelength is 632.8 nm. The running buffers were HEPES, and PBS solutions.

To show the significant advantages of TBG superlattice compared with monolayer graphene and bilayer graphene without relative twisting, four important indicators employed for describing SPR effect such as minimum reflectivity, incident angle (SPR angle), phase, and GH shift were studied in detail. As shown in Figure 2(a), when the twisted angle was located at 55.3°, 44 nm Au film-1-TBG hybrid configuration exhibited the smallest minimum reflectivity of 2.2038 × 10^−9^ in two buffers including HEPES and PBS solutions.

In contrast, the minimum reflectivity of other two hybrid configurations: 44 nm Au film-monolayer graphene, 44 nm Au film-bilayer graphene without any twisting, was 0.0032 and 9.8415 × 10^−5^, respectively. It indicates that, 44 nm Au film-1-TBG model with a twisted angle of 55.3° has the largest photon absorption, and it can provide the strongest SPR enhancement. Meanwhile, it also has been confirmed that, twisting operation can produce significant effects on photon absorption. In addition, the optimal incident angles corresponding to 44 nm Au film-monolayer graphene, 44 nm Au film-bilayer graphene without any twisting, and 44 nm Au film-1-TBG model with a twisted angle of 55.3° was 54.879°, 55.0769°, and 54.9151°, respectively. It indicated that, there was no obvious difference in SPR angle. Figure 2(b) showed significant phase jump behavior based on three SPR models above mentioned. Interestingly, the point of phase jump is identical to the dip of minimum reflectivity. Moreover, the 44 nm Au film-1-TBG model with a twisted angle of 55.3° showed the sharpest jump because of strongest SPR gain. More interestingly, the obtained GH shift showed sharper jump behavior than phase, as depicted in Figure 2(c). The GH shift derived from 44 nm Au film-monolayer graphene was 53.8606 µm, and the GH shift generated from 44 nm Au film-bilayer graphene without any twisting was −263.9574 µm. However, a huge GH shift of 4.7485 × 10^4^ µm was obtained by twisting a planar relative angle (55.3°) of 44 nm Au film-1-TBG model. It indicated that, GH shift can be further enlarged by SPR enhancement. The comparison of minimum reflectivity, phase, and GH shift generated from 44 nm Au film-1-TBG with a twisted angle of 55.3° was depicted in Figure 2(d). When the running buffer was changed to be Trizma solution, the obtained minimum reflectivity, phase, and GH shift generated by 44 nm Au film-1-TBG with a twisted angle of 55.3° were shown in Figure S1. The obtained largest changes in differential phase and GH shift were 85.8041° and −3838.3 µm, respectively. Compared to minimum reflectivity and phase, GH shift is a more sensitive indicator to evaluate biosensing ability of our proposed plasmonic biosensor.

**Figure 3:**
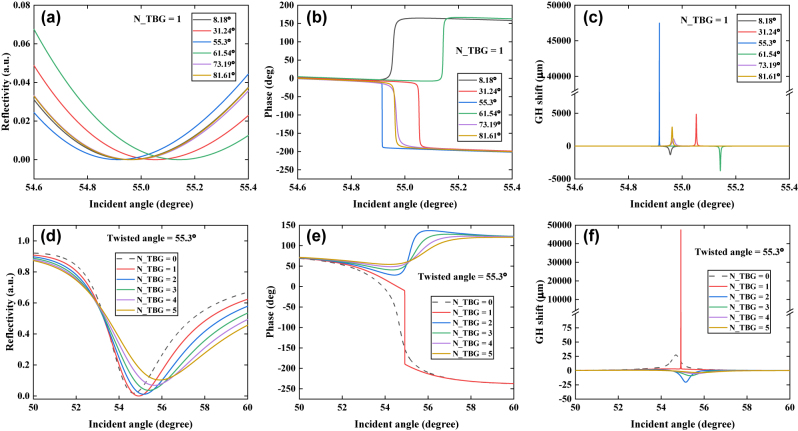
Variation in minimum reflectivity (a), phase (b) and GH shift (c) via varying the twisted angle of 1-TBG system (8.18°, 31.24°, 55.3°, 61.54°, 73.19°, and 81.61°). Noting that, the number of TBG system is fixed at 1. The thickness of Au film is 44 nm, and the excitation wavelength is 632.8 nm. Variation of minimum reflectivity (d), phase (e) and GH shift (f) by varying the number of TBG system (0–5). Noting that, the twisted angle of TBG is fixed at 55.3°. The thickness of Au film is 44 nm, and the excitation wavelength is 632.8 nm. N_TBG stands for the number of TBG layer. The running buffers were HEPES, and PBS solutions.

It is possible that, twisting can provide a new approach to tune the photon absorption of a TBG system. Next, both twisted angle and thickness of TBG system was optimized to study the SPR effects, as shown in Figure 3, Figure S2, and Table 2. Both Figure 3(a)–(c) and Table 2 clearly showed that, the variation in twisted angle (8.18°, 31.24°, 55.3°, 61.54°, 73.19°, and 81.61°) could produce significant changes in minimum reflectivity, phase, and GH shift. For example, the TBG system with a twisted angle of 8.18° could produce a reflectivity of 4.38 × 10^−6^ (black curve, Figure 3(a)). Afterwards, the reflectivity could be further lowered by an order of magnitude to be 3.07 × 10^−7^ when a larger twisted angle was tuned to be 31.24° (red curve, Figure 3(a)). Undoubtedly, the best photon absorption condition occurred as twisted angle was tuned to be 55.3°, resulting in an ultra-low reflectivity of 2.2038 × 10^−9^ (blue curve, Figure 3(a)). Accompanied by Heaviside step-like phase jumps (Figure 3(b)), the enhanced GH shifts (Figure 3(c)) corresponding to twisted angle at 8.18°, 31.24°, 55.3°, 61.54°, 73.19°, and 81.61° were −1309.26, 4851.113, 47,485, −3760.17, 1099.598, and 2905.688 µm, respectively.

**Table 2:** Reflectivity and GH shift based on various twisted angles and number of TBG superlattice.

Twisted angle	Number of TBG layer	Reflectivity (a.u.)	GH shift (µm)
8.18°	1	4.38 × 10^−6^	−1309.26
	2	0.01208	−19.2709
	3	0.03912	−8.4699
	4	0.07328	−4.9940
	5	0.11032	−3.344
31.24°	1	3.07 × 10^−7^	4851.113
	2	0.01123	−19.1981
	3	0.03742	−8.1436
	4	0.07098	−4.6687
	5	0.10774	−3.0471
55.3°	1	2.2038 × 10^−9^	47,485
	2	0.01112	−20.4043
	3	0.03649	−8.9857
	4	0.06855	−5.3386
	5	0.10323	−3.6059
61.54°	1	4.87 × 10^−7^	−3760.17
	2	0.01173	−17.9426
	3	0.03869	−7.4745
	4	0.07319	−4.1954
	5	0.11098	−2.68104
73.19°	1	6.27 × 10^−6^	1099.598
	2	0.01049	−20.8318
	3	0.03554	−8.9676
	4	0.06778	−5.2438
	5	0.10313	−3.4919
81.61°	1	9.13 × 10^−7^	2905.688
	2	0.01119	−20.5184
	3	0.03752	−8.9380
	4	0.07143	−5.2622
	5	0.10867	−3.5229

In addition, the number of stacked TBG system also played an important role in achieving strongest SPR enhancement, as shown in Figure 3(d)–(f). When the number of TBG system ranged from 0 to 5, minimum reflectivity, phase jump, and GH shift were obviously different, as listed in Table 2. In the absence of TBG, 44 nm Au film showed a reflectivity of 0.01796 (black dashed curve, Figure 3(d)). However, the photon absorption could be greatly improved to be 2.2038 × 10^−9^ by introducing a TBG system. However, when the number of TBG system was no less than 2, the obtained reflectivity was gradually increased, and the incident angle was red-shifted (Figure 3(d)). It indicates that, the balance between photon absorption and energy loss was broken via further introducing excess TBG system. The energy loss dominated in such conditions, and it needed to larger incident angle to excite SPR. With increasing TBG systems, the phase transition Figure 3(e) changed from positive value to negative value, which was synchronized with GH shift. When the number of TBG (twisted angle at 55.3°) was from 0 to 5, the obtained GH shift (Figure 3(f)) was 22.3401, 4.7485 × 10^4^, −20.4043, −8.98569, −5.3386, and −3.60594 µm, respectively. In all, the best plasmonic configuration: 44 nm Au film/1-TBG with a twisted angle of 55.3°, could achieve ultra-high detection sensitivity by measuring GH shift.

**Figure 4:**
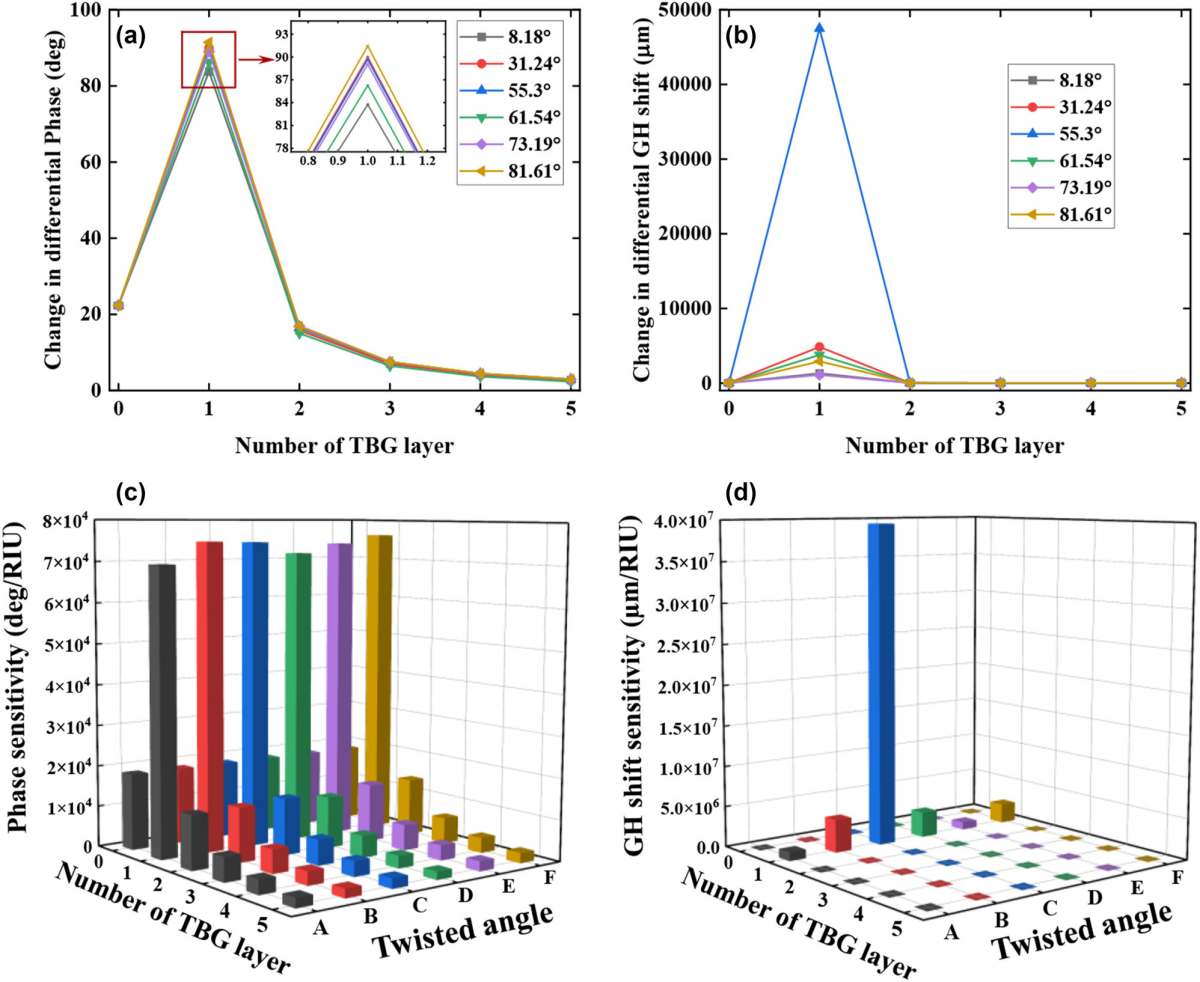
Change in differential phase (a) and differential GH shift (b) by varying the number of TBG system (0–5) and twisted angle (8.18°, 31.24°, 55.3°, 61.54°, 73.19°, and 81.61°) due to a defined RI variation of 0.0012 RIU. The thickness of Au film is 44 nm, and the excitation wavelength is 632.8 nm. Calculated phase (c) and GH shift (d) sensitivity by varying the number of TBG system (0–5) and twisted angle of TBG (8.18°, 31.24°, 55.3°, 61.54°, 73.19°, and 81.61°). Notes: The twisted angles of A–F in Figure 4(c) and (d) stand for the angles at 8.18°, 31.24°, 55.3°, 61.54°, 73.19°, and 81.61°, respectively. The running buffers were HEPES, and PBS solutions.

It has been proposed that, a RI variation of 0.0012 RIU was generated by the adsorption interaction between 1 picomole (pM) bioanalytes with molecular weight less than 8 kDa and sensing interface [35]. For a defined RI variation (Δ*n*_bio_ = 0.0012 RIU) generated by adsorption interactions, changes in differential phase (Δ*φ*_*d*_) and GH shift (Δ*GHS*_*d*_) were plotted to test the biosensing ability of our proposed biosensor, as shown in Figure 4(a) and (b). For various thickness of TBG, the obtained differential phase was significantly different. For instance, the largest change in differential phase (yellow curve, Figure (4a)) was 91.4451°, obtained from such configuration: 44 nm Au film/1-TBG with a twisted angle of 81.61°. However, when the number of TBG was more than 1, the obtained change in differential phase decreased significantly. Conversely, for various twisted angles, there was no significant difference in differential phase. For instance, when the number of TBG system was fixed at 1, the largest change in differential phase for twisted angle at 8.18°, 31.24°, 55.3°, 61.54°, 73.19°, and 81.61° is 83.7510°, 89.9945°, 89.6786°, 86.2708°, 89.0303°, and 91.4551°, respectively. Therefore, the 44 nm Au film/1-TBG configuration under these six twisted angles (8.18°, 31.24°, 55.3°, 61.54°, 73.19°, 81.61°) exhibited almost equivalent detection sensitivity, as shown in Figure 4(c).

In addition, change in SPR angle (*θ*_SPR_) was also studied, as shown in Figure S3. It can be found that, the introduction of TBG system cannot produce positive function on improving photon absorption. The red-shifts generated by the configuration: 44 nm Au film/1-TBG with a twisted angle of 55.3° was only as small as 0.042°. In comparison, the change in differential GH shift was much larger than both differential phase and SPR angle, as shown in Figure 4(b). The reason is that, GH shift originated from the derivative of phase versus incident angle, and the tiny difference in phase modulation can be further amplified by GH shift modulation. As predicted, the change in differential GH shift obtained by various twisted angles showed significant difference rather than tiny variation in differential phase. For example, when the twisted angle (*α*) is 8.18°, 31.24°, 55.3°, 61.54°, 73.19°, and 81.61°, the obtained change in differential GH shift is 1309.26 µm, 4851.113 µm, 47,485 µm, 3760.17 µm, 1099.598 µm, and 2905.688 µm, respectively. Obviously, the configuration: 44 nm Au film/1-TBG with a twisted angle of 55.3° can provide the first largest change in differential GH shift of 4.7485 × 10^4^ µm (blue curve). It has been reported that, under the excitation of 632.8 nm, a gold-Ge_2_Sb_2_Te_5_(GST) metasurface with a theoretical GH shift of 2107 µm was confirmed to have the ability of excellent ultrasensitive detection for cancer biomarkers [45]. Although the value of first largest GH shift is much larger than other values of GH shift obtained by 31.24°, 61.54°, and 81.61°, the three latter still have the ability to perform ultrasensitive detection of targeted bioanalytes. Considering the largest change in differential GH shift of 4.7485 × 10^4^ µm, the largest GH shift detection sensitivity was as high as 3.9570 × 10^7^ µm/RIU, as depicted in Figure 4(d). Compared with other reported 2D plasmonic configurations modulated by GH shift (Table 3), 44 nm Au film/1-TBG heterostructure exhibited higher GH shift detection sensitivity. It is worth pointing out, although our proposed configuration 44 nm Au film/1-TBG could provide an ultra-high GH detection sensitivity of 3.9570 × 10^7^ µm/RIU, it also exhibited tunable performance by changing different twisted angles. To achieve ultrasensitive detection of targeted bioanalytes, one can choose phase modulation using 44 nm Au film/1-TBG with six tunable twisted angles at 8.18°, 31.24°, 55.3°, 61.54°, 73.19°, and 81.61°. When it switched to GH shift modulation, one can choose 44 nm Au film/1-TBG with four tunable twisted angles at 31.24°, 55.3°, 61.54°, and 81.61°.

**Table 3:** Comparison of 2D plasmonic configurations under the excitation of 632.8 nm.

Configuration	Sensitivity (µm/RIU)	Change in RI	Ref.
Au/MoS_2_/graphene	3.509 × 10^5^	0.002	[46]
Au/ITO/MoSe_2_/graphene	5.075 × 10^5^	0.002	[30]
Cu/BlueP/WS_2_/graphene	2.024 × 10^6^	0.0002	[31]
Au/TBG	3.9570 × 10^7^	0.0012	Current work

For a RI variation of 0.0012 RIU, our proposed configuration has a GH shift detection sensitivity of 3.9570 × 10^7^ µm/RIU. It means that, the theoretical largest GH shift was determined to be 4.4785 × 10^4^ µm. Although there is no experimental result to support such high GH shift sensitivity, our proposed Au/1-TBG configuration still has great feasibility in performing ultrasensitive biosensing applications. In addition, other reported 2D plasmonic metasurfaces have shown ultrasensitive biosensing ability using GH detection. For theoretical proposals, a 2D perovskite-enhanced plasmonic configuration provided a theoretical record-high value of 1.2862 × 10^9^ µm/RIU [43]. More recently, an optimal gold film-bismuth selenide (Bi_2_Se_3_)-graphene metasurface could provide an ultra-high GH shift sensitivity of 8.5017 × 10^6^ µm/RIU [34]. For experimental observations, a groove hyperbolic metasurface with a theoretical largest GH shift sensitivity of 1.0134 × 10^4^ µm/RIU, can perform ultrasensitive biosensing performance [47]. For large molecule weight analytes such as bovine serum albumin (BSA), the detection limit was determined to be 1 × 10^−19^ mol/L. For low molecule weight analytes such as biotin, the detection limit was measured to be 1 × 10^−15^ mol/L. Recently, a gold-GST metasurface was successfully fabricated to perform ultrasensitive detection of cancer biomarkers [45]. The calculated largest GH shift of GST-gold metasurface was 2107 µm, but experimental observation of actual GH shift was 341.9 μm. It also can be found that, there was difference in value of GH shift between theoretical prediction and experimental measurement. More importantly, the obtained detection limit of cancer marker tumor necrosis factor-alpha was determined to be 1 × 10^−15^ mol/L.

**Figure 5:**
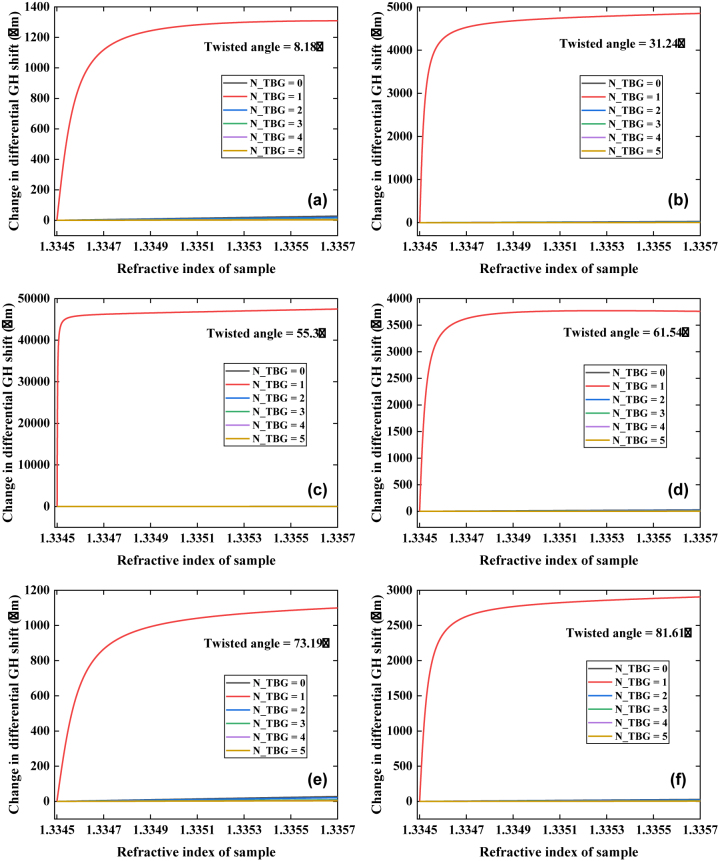
Change in differential GH shift versus RI variation in sensing interface of Au film-TBG configuration by tuning the number (0–5) of TBG system and twisted angles: (a) 8.18°, (b) 31.24°, (c) 55.3°, (d) 61.54°, (e) 73.19°, and (f) 81.61°. Noting that, the thickness of Au film is 44 nm, and the excitation wavelength is 632.8 nm. N_TBG stands for the number of TBG layer. The running buffers were HEPES, and PBS solutions.

The changes in differential GH shift with respect to the RI of sensing interface from 1.3345 to 1.3357 RIU was investigated by changing the number of TBG system (0–5) and twisted angles (8.18°, 16.51°, 25.19°, 31.24°, 55.3°, 61.54°, 73.19°, and 81.61°), as shown in Figures 5 and S4. For each twisted angle, the change in differential GH shift generated by 44 nm Au film/1-TBG system exhibited significant response to record the RI change in sensing interface. In particular, when the twisted angle of TBG was 55.3°, the differential GH shift response was steepest, as depicted in Figure 5(c). It indicates that, 44 nm Au film/1-TBG (twisted angle of 55.3°) can achieve ultra-sensitive biosensing. In addition, when the number of TBG system was more than 1, it showed a stable linear relationship between the change in differential GH shift and RI variation in Au film-1-TBG sensing interface. However, these calculated GH shifts were extremely weak, and they are not suitable to perform ultrasensitive biosensing. It can be found that, one configuration: 44 nm Au film-1-TBG (twisted angle at 55.3°) produces the strongest GH shift response, exhibiting great promising for ultrasensitive detection of bioanalytes.

**Figure 6:**
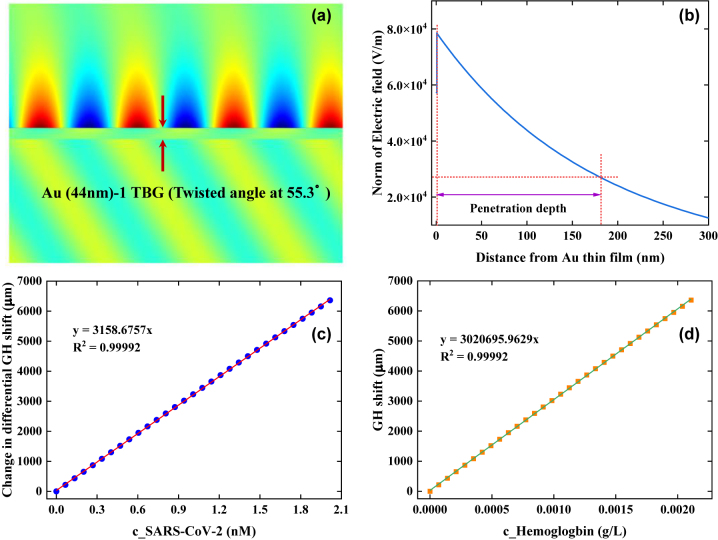
Highly enhanced evanescent field in 44 nm Au film-1 TBG hybrid configuration employed for quantitatively monitoring bioanalytes. (a) Generation of significantly enhanced evanescent field at the sensing interface of 44 nm Au film-1 TBG hybrid configuration. (b) Decay behavior of evanescent field propagating into running buffer. Excellent linear relationship between detection concentration of SARS-CoV-2 (c) and hemoglobin (d) and changes in differential GH shift in the configuration: 44 nm Au film-1-TBG with a twisted angle at 55.3°. Dotted curves in Figure 6(c) and (d) stand for demonstration from linear fitting.

Prior to achieving quantitatively monitoring the concentrations of targeted bioanalytes, extracting a linear response interval based on the optimal configuration (44 nm Au film-1-TBG [twisted angle at 55.3°]) is necessary. Figure S5 demonstrated that, there was a linear GH shift response for an extremely tiny RI change as little as 10^−7^ RIU. Compared with 44 nm Au film and 44 nm Au film-bilayer graphene without any twisting, our proposed plasmonic configuration: 44 nm Au film-1-layer TBG (twisted angle of 55.3°) could provide an enhancement factor (EF) of 9.1 × 10^5^, and 3.2 × 10^3^, respectively. To figure out the reason for obtaining high gain, an enhanced evanescent field in the 44 nm Au film-1-TBG (twisted angle at 55.3°) configuration was plotted, as shown in Figure 6(a). When the excitation wavelength was 632.8 nm, a hugely enhanced evanescent field was regularly generated at sensing interface of TBG superlattice. Furthermore, the evanescent field intensity reduces exponentially in the direction of running buffer layer, and the penetration depth is approximately 180 nm (Figure 6(b)).

Considering as the linear response interval of 44 nm Au film-1-TBG hybrid configuration, theoretical feasibility of linearly detecting SARS-CoV-2 and human hemoglobin was studied. The molecular weight of individual SARS-CoV-2 was approximately 800 kDa [48]. Based on Equation (2), molecular weight of targeted bioanalytes only affected the theoretical detection concentration, and it cannot change the ultrasensitive detection ability of 44 nm Au film-1-TBG hybrid configuration. Thus, a linear relationship between change in differential GH shift (µm) and detection concentration of SARS-CoV-2 was studied. For an extremely tiny RI variation of 3 × 10^−7^ RIU, an almost perfect linear detection section for SARS-CoV-2 was from 0 to 2 nM (Figure 6(c)), which can be described by an equation Δ*GHS*_*d*_ = 3158.6757*c_SARS_ − CoV − 2, where *c*_SARS_ − CoV − 2 denotes the concentration of SARS-CoV-2. In addition, 44 nm Au film-1-TBG hybrid configuration also showed an excellent linear response interval from 0 to 0.002 g/L for human hemoglobin. The linear response can be described by an equation Δ*GHS*_*d*_ = 3,020,695.9629*c_Hemoglobin_, where *c*_Hemoglobin_ denotes the concentration of human hemoglobin. Here, we believe that, our proposed plasmonic configuration has great potential for quantitatively monitoring SARS-CoV-2 and human hemoglobin.

## 4 Conclusions

In this study, a sensitivity hugely enhanced SPR biosensor was proposed via introducing a TBG superlattice. In brief, our proposed plasmonic biosensor was composed of a plasmonic Au film, and a TBG superlattice. Interestingly, our proposed plasmonic biosensor showed tunable biosensing ability, highly depending on the planar twisted angle of TBG superlattice (*α*). Moreover, the photon absorption efficiency in Au film-TBG heterostructure can be significantly improved by tuning the twisted angle. Compared to phase modulation, GH shift is superior to achieving ultrasensitive biosensing. When the wavelength of incident light is 632.8 nm, the best configuration: 44 nm Au film/1-TBG (twisted angle at 55.3°) heterostructure, can produce an ultralow reflectivity of 2.2038 × 10^−9^ and largest GH shift of 4.4785 × 10^4^ µm. For a fixed RI variation of 0.0012 RIU, 44 nm Au film/1-TBG (twisted angle at 55.3°) heterostructure could produce an extremely high GH sensitivity of 3.9570 × 10^7^ µm/RIU. In addition, our proposed biosensor also exhibited theoretical operability of quantitatively detecting SARS-CoV-2 and human hemoglobin via extracting a linear RI variation as small as 3 × 10^−7^ RIU. For SARS-CoV-2, a good linear detection section was determined from 0 to 2.0 nM. For human hemoglobin, a linear detection range was from 0 to 0.002 g/L. It can be foreseen that, our proposed SPR biosensor is ideal candidate for quantitatively detecting SARS-CoV-2 and human hemoglobin in biomedical applications.

## Supplementary Material

Supplementary Material Details
