# Supplementary material for: Sensitivity enhanced tunable plasmonic biosensor using two-dimensional twisted bilayer graphene superlattice
Source: Nanophotonics. 2023 Mar 7;12(7):1271–84. doi: 10.1515/nanoph-2022-0798 (PMC11636415; doi:10.1515/nanoph-2022-0798)
Supplement: Supplementary file 1 — Supplementary Material Details [file j_nanoph-2022-0798_suppl_001.docx]

**Sensitivity Enhanced Tunable Plasmonic Biosensor Using Two-Dimensional** **Twisted Bilayer Graphene Superlattice**

Fusheng Du ^1^, Kai Zheng ^2^, Shuwen Zeng ^3*^, and Yufeng Yuan^1 *^

^1^ School of Electronic Engineering and Intelligentization, Dongguan University of Technology, Dongguan, 523808, China

^2^ School of Civil Aviation, Northwestern Polytechnical University, Xi’an, Shanxi, 710072, China

^3^ Light, Nanomaterials & Nanotechnologies (L2n), CNRS-ERL 7004, Université de Technologie de Troyes, 10000 Troyes, France

* Correspondence: yufengyuan@dgut.edu.cn (Y.Y.), shuwen.zeng@cnrs.fr (S.Z.)

**Supplementary Figures**

**Figure S1.** (a) Comparison of minimum reflectivity (blue), phase (red) extracted from Au film-1-TBG with a twisted angle of 55.3^°^. (b) GH shift extracted from Au film-1-TBG with a twisted angle of 55.3^°^. Change in differential phase (c) and differential GH shift (d) obtained from Au film-1-TBG with a twisted angle of 55.3^°^. Noting that, the thickness of Au film is 44 nm, and the excitation wavelength is 632.8 nm. The running buffer is Trizma solution.

**Figure S2.** Variation in minimum reflectivity (a), phase (b) and GH shift (c) via varying the twist angle of TBG system (8.18^°^, 31.24^°^, 61.54^°^, 73.19^°^, and 81.61^°^). Noting that, the number of TBG system is fixed at 1. Noting that, the thickness of Au film is 44 nm, and the wavelength of incident light is 632.8 nm. N_TBG stands for the number of TBG layer. The running buffers are HEPES, and PBS solutions.

**Figure S3.** Change in incident angle (a) and obtained SPR angle detection sensitivity (b) by varying the number of TBG systems and twisted angle of TBG for a defined RI variation of 0.0012 RIU. Noting that, the thickness of Au film is 44 nm, and the wavelength of incident light is 632.8 nm. The running buffers are HEPES, and PBS solutions.

**Figure S4.** Change in differential GH shift with respect to a tiny RI variation (0.0012 RIU) by changing the number of TBG systems (0-5) and twisted angles of TBG: (a) 16.51° and (b) 25.19°. Noting that, the thickness of Au film is 44 nm, and the wavelength of incident light is 632.8 nm. The running buffers are HEPES, and PBS solutions. N_TBG stands for the number of TBG layer.

**Figure S5.** Comparison of GH-based biosensing performance generated by 44 nm Au film, 44 nm Au film-bilayer graphene without twisting, and 44 nm Au film deposited with 1-TBG (twisted angle is 55.3^°^) for a tiny RI variation, as low as 10^-7^ RIU. Noting that, the running buffers are HEPES, and PBS solutions.
